# Exploration of association rule mining between lost-linking features and modes of loan customers using the FP-growth algorithm for risk warning strategies

**DOI:** 10.1371/journal.pone.0332623

**Published:** 2025-09-23

**Authors:** Jiaqi Wang, Xiaolong Jiang, Yizhou He, Biyu Guan, Chao Deng

**Affiliations:** 1 Faculty of Logistics, Guangdong Mechanical & Electrical Polytechnic, Guangzhou, China; 2 Macao Polytechnic University, Macao, China; 3 School of Management, Jinan University, Guangzhou, China; 4 Guangdong University of Science and Technology, Dongguan, China; 5 Guangdong Rural Credit Union, Guangzhou, China; Southwest Petroleum University, CHINA

## Abstract

In the new model of China’s dual-circulation economy, the opening-up and deepening of financial markets have imposed higher requirements on the risk management capacity of financial institutions, with the issue of loan customers losing contact and defaulting becoming an urgent concern. Based on desensitized samples of lost-linking customers (with multidimensional features such as communication behavior and loan qualifications), this study uses the FP-Growth algorithm to systematically mine association rules between loss-of-contact features and three modes: “Hide and Seek”, “Flee with the Money”, and “False Disappearance”, providing effective risk management strategies for financial institutions. Through association rule mining, this study reveals significant correlations between some feature combinations and lost-linking modes. The results reveal substantial variations in correlation strength among different feature combinations and lost-linking modes, and the association strength increases significantly with the prolongation of overdue time. The results provide banks with quantitative early warning signs based on feature combinations, which can be applied to risk-grading monitoring systems. The research emphasizes the requirement for combined analysis of multidimensional features and dynamic monitoring in precise risk control.

## 1. Introduction

With China’s new development paradigm, emphasizing an economy centered domestically with complementing domestic and global cycles, deepening and opening up of financial markets have imposed higher requirements on the risk management role of financial institutions. In particular, loan customers’ lost-linking and defaults are significant issues. The phenomenon of loan customers losing contact not only reduces the loan recovery rate, leading to direct economic losses for financial institutions, but also may trigger a chain reaction that exerts a negative impact on the stability and healthy development of the financial market [1]. As the loan market has been growing continuously, the phenomenon of loan customers losing contact has become increasingly prevalent. Traditional single-indicator risk management tools are ineffective in addressing strategic lost-linking behaviors, such as abusing their mortgage loan qualifications to evade debt in the “Flee with the Money” mode. Banks must urgently determine the lost-linking aspects of loan customers and predict the lost-linking modes, thereby reducing risks through the application of a scientific method. Therefore, the accurate recognition of lost-linking features and modes has come to be a pressing issue that financial institutions must counteract.

Following the trend in finance research, a number of studies have attempted to investigate loan lost-linking data from various perspectives to identify their behavioral patterns. Such researches often utilize data obtained from customers’ social communication data, common consumption transaction data, and loan transactions on financial platforms. For instance, [2] proposed an information matching model and multi-perspective tracking algorithms, using mobile social big data to extract contact information of defaulting loan customers and providing a framework for this paper to dig out attributes of defaulting loan customers from multi-source data. [3] developed a path association index model and network sorting search algorithms based on loan customers’ daily consumption transaction data, successfully identifying transaction behavior patterns over a specific period. [4] is based on a multi-agent Q-Learning collaborative search model that integrates social, e-commerce, and financial platform data to identify information on default borrowers, emphasizing the necessity of multi-platform data fusion in lost-linking customer management, presenting implications for the holistic use of data in this research. These studies have played a significant role in identifying the social relationships, daily behavioral trajectories, and multi-platform information related to loan customers who have lost contact in the financial sector—findings that provide valuable references for financial risk management. Nevertheless, existing works mainly focused on single feature prediction or general default prediction (e.g., taking into account only the days overdue). Few studies systematically explore the relationships between typical evasion patterns—such as “Hide and Seek” (keeping active mobile phone numbers but avoiding contact), “Flee with the Money” (escaping with borrowed funds), and “False Disappearance” (blocking emergency channels to fake absence)—and borrowers’ multidimensional features. This gap hinders the identification of strategic differences in borrowers’ deliberate debt-avoidance behaviors. Unlike previous studies that focus on single features or general default indicators (e.g., only overdue duration), we explicitly mine associations between multidimensional features and three strategic evasion patterns: “Hide and Seek”, “Flee with the Money”, and “False Disappearance”.

In the interest of describing the intrinsic interconnection between lost-linking features and lost-linking modes, this study suggests the FP-Growth algorithm on desensitized lost-linking customer data. It algorithmically digs up association rules among loan customer lost-linking features and the three typical lost-linking modes and illustrates the strong predictability of feature combinations for a specific lost-linking mode. The goal is to provide financial institutions with greater risk management methods and decision support for dealing with loan lost-linking customers.

The most critical part of being able to effectively determine lost-linking attributes’ relation to lost-linking modes is frequent itemset and association rule mining of lost-linking attributes and lost-linking modes, typically through the use of effective association rule mining algorithms. For instance, the FP-Growth algorithm discussed in [5] constructs an FP-Tree data structure that requires only two passes over the database and minimizes data reads to make the algorithm very efficient. The essence of this approach is to correctly identify the frequent itemsets of lost-linking features and extract association rules statistically significant, thus uncovering the intricate association between lost-linking features and modes. This requires not only the algorithm’s efficiency and accuracy but also careful preprocessing of the data and selection of features.

However, this method is confronted with three main challenges: (1) High complexity of data processing: Lost-linking datasets typically contain a combination of feature types, i.e., both continuous and discrete features. The features must undergo preprocessing prior to applying association rule mining to ensure data consistency and the effectiveness of the algorithm. (2) Feature selection and detection of association patterns: Among the myriad lost-linking features, determination of which of them significantly contribute to the revelation of lost-linking modes and mixes that can form semantic association rules is a long process involving rigorous data analysis and digging. Feature selection rationale and accuracy will have a direct bearing on the outcome of association rule mining. (3) Algorithm parameter tuning and result interpretation: The parameter settings in association rule mining algorithms, i.e., the minimum support threshold and minimum confidence threshold, significantly affect the mined results. How to make suitable adjustments to these parameters based on actual needs and data characteristics is an issue that must be well addressed. Besides, the identified association rules should be explained and verified to hold and be effective in real-life application.

In order to describe the correlation between lost-linking features and modes, this study applies the FP-Growth algorithm to examine the lost-linking features of loan lost-linking customers and concealed relationship rules and modes. Based on the new development environment, research in the management of loan lost-linking customers is becoming more and more popular. As an efficient association rule mining technique, the FP-Growth algorithm has shown extensive potential in financial risk management. Several studies in recent years have investigated the use of the FP-Growth algorithm and its variants in discovering financial risk features. For example, [6] proposed a hybrid approach that combines the FP-Modified Tree and Apriori Growth mining algorithms to detect credit card fraud. This research is critical for understanding how to optimize FP-Growth-related algorithms for financial risk detection scenarios, which in turn enables the identification of risk features in the management of loan lost-linking customers. [7] proposed a hybrid system based on the FP-Tree and Apriori algorithms for weighted rule mining on transaction datasets. They also emphasized that data description, exploration, and selection are key phases in the data mining process, and these phases precisely correspond to the risk feature identification phase in the management of lost-linking customers, both of which require extracting useful patterns from large amounts of data and constructing models to predict and identify potential lost-linking risks. [8] used discretization and FP-Growth association rule mining techniques to identify abnormal patterns in complex financial transactions, which is one technique that has enjoyed exponential success in financial anti-fraud studies. [9] combined clustering algorithms with feature engineering and frequent itemset extraction techniques to perform multidimensional analysis of financial transaction information, which enhances the ability to identify abnormal trading patterns in the financial market. Such multi-technology integration system provides an integrated data processing and analysis system for loan lost-linking customer management, which supports the identification of hidden patterns and risk signals of loan lost-linking from complex financial transaction data, and possesses enough support for early warning and management of loan lost-linking customers.

Overall, these studies indicate that FP-Growth algorithm and its extensions possess great potential for application in the field of financial risk management.

In the field of empirical research, application of the FP-Growth algorithm in customer management has yielded remarkable results. [10] created a Three-Dimensional Fuzzy FP-Tree to conduct temporal data mining, conducting empirical research on customer transaction data and effectively solving dynamic change of time series data, thereby bringing about new concepts and approaches for time series data analysis in financial risk management. [11] used the FP-Growth algorithm and ARIMA and LSTM models to analyze sales and market demand information. Extracting frequent itemsets and association rules, it uncovered the complexity of shopping behavior of customers and offered lending institutions opportunities for risk management. [12] presented a new incremental maximum frequent itemset mining algorithm that considers subjective interest criteria during mining and was implemented and tested on public databases with results indicating its effectiveness in handling incremental data and reducing the production of uninteresting rules by a large margin. [13] applied the RFP-Growth algorithm for region-based association rule mining in IoT networks, analyzing transaction records with spatial data to identify some patterns of association among regions and thereby provide robust supporting data for inventory management and price strategy in retail enterprise. Such a method aids a more precise understanding of the behavior patterns of different areas of customers to develop targeted strategies for managing loan lost-linking customers and enable financial institutions to resource more efficiently and optimize the efficiency of risk regulation.

These works further enriched the theory and practice of the FP-Growth algorithm and employed technical approaches to further apply the FP-Growth algorithm to mine the association rules between lost-linking attributes and channels of loan lost-linking clients. Though some success has been found in managing risk of loan defaults and lost-linking customers, prior research has not yet unraveled profoundly the customer evasion strategies in the “Hide and Seek” mode (concealing mobile phone numbers in network but making no contact), the malicious evasion behavior rationale using mortgage loan eligibility in the “Flee with the Money” mode, or the strategic differences in fake disappearance through sabotage of emergency contact channels in the “False Disappearance” mode. In the context of the new development paradigm and the mutually reinforcing dual circulation, financial institutions are in immediate need of accurate identification tools appropriate to these differentiated lost-linking modes to counter customers’ advanced debt evasion strategies.

In this paper, our goal is to investigate the connection between lost-linking properties and lost-linking customer modes of loans in the new development paradigm. To achieve this, we suggest employing the FP-Growth algorithm, a compact data mining technique for the association rule mining problem, to conduct an intensive study of the lost-linking data to find implicit frequent itemsets and association rules. With this research, we hope to reveal the intrinsic complex factors of loan customer disconnection and their interactive mechanisms, particularly the difference of customer characteristics in dimensions such as address authenticity, communication styles, and loan type under different lost-linking modes, to provide financial institutions with more precise risk warnings and management recommendations.

The contribution of this study is applying the FP-Growth algorithm to deeply excavate frequent itemsets and association rules from the lost-linking dataset, revealing for the first time the systematic strong correlation among lost-linking modes (e.g., “Hide and Seek”, “Flee with the Money”, and “False Disappearance”) and multidimensional features. These are the “mobile phone number available but unreachable + long-term high overdue” combination’s predictability for the “Hide and Seek” mode, the indicative nature of “business address + mortgage loan features” for the “Flee with the Money” mode, and the association of “emergency contacts lost-linking + short-term lost-linking” with the “False Disappearance” mode. These findings not only provide additional information and evidence to lenders on disconnection risks but also help to form more correct and efficient risk management methods.

Besides, this paper also proposes developing a data analysis-based risk early warning system that is able to set up quantitative indicators based on the combination of lost-linking properties and importance of association rules, auto-generating risk warnings and providing real-time risk management services for lending institutions. This framework is valuable reference for risk management practices in financial institutions.

The rest of the paper is organized as follows. Question description section gives the question description. Methodology section presents the FP-Growth algorithm’s principles and procedure. Model Application section applies the FP-Growth algorithm to empirical analysis, demonstrates the mining effect of the algorithm on lost-linking datasets, and carries out deep analysis and discussion of the mining results. Risk Early Warning System Architecture Design section introduces the risk early warning system architectural design systematically. Conclusions section concludes this paper.

## 2. Question description

The loan lost-linking customers investigated in this paper are those who lose contact with lending institutions such as banks, microfinance companies or financial platforms before they have repaid their debts during the project implementation period or by the due date of repayment [14]. Lost-linking modes refer to the fundamental ways in which loan customers deliberately create the situation of losing contact with some relevant personnel with the intention of evading the repayment of debts to lending institutions.

This study employs the FP-Growth algorithm to investigate the lost-linking features of loan lost-linking customers and the underlying association rules and patterns. It primarily covers the following three lost-linking modes:

The Hide and Seek mode (HS mode) is characterized by evasive actions by loan customers under financial stress or inclined to avoid debt, such as delayed repayments, intentional device shutdowns, refusal to answer calls, and frequent changes of residence, thereby creating an illusion of unreachability for lending institutions while maintaining limited contact possibilities, such as keeping their mobile phone numbers active.

The Flee with the Money mode (FM mode) refers to scenarios where loan customers, after obtaining a large loan, sever all communication channels with lending institutions, deactivating or closing all known contact methods, and taking extreme measures such as fleeing their usual place of residence with the loaned funds to completely evade debt recovery, demonstrating a clear intention to maliciously avoid debt.

The False Disappearance mode (FD mode) involves loan customers who intentionally fabricate a state of disappearance to evade debt, by deactivating their mobile phones, canceling their numbers, leaving their usual place of residence and workplace, and severing all social ties to conceal their whereabouts, misleading lending institutions into incorrectly assessing them as missing. This behavior exhibits a high degree of avoidance intent and strategic thinking on the part of the customers, posing a significant challenge to lending institutions in debt collection. These three lost-linking modes are refined based on practical data from bank risk control practices, including post-loan tracking records, debt collection logs, and customer behavior analysis in actual lending scenarios, ensuring alignment with real-world debt evasion patterns observed by financial institutions.

The aforementioned three lost-linking modes represent common debt evasion strategies employed by loan customers, providing valuable reference for financial institutions in assessing the lost-linking status and debt evasion intentions of loan customers [15,16]. Consequently, an in-depth exploration of the association between lost-linking features and lost-linking modes among loan lost-linking customers holds significant importance for enhancing the risk management efficiency of financial institutions. To effectively address this issue, this study is dedicated to resolving the following key questions:

(1) How to identify key features with strong predictive power for the three modes (“Hide and Seek”, “Flee with the Money”, “False Disappearance”) from multidimensional features, including communication behavior (mobile phone status, emergency contact reachability) and loan qualifications (overdue days, overdue amount)?

Loan lost-linking customers exhibit multiple lost-linking features, such as permanent address, mobile phone number status, loan-related information, etc. However, among these numerous features, some may have a decisive impact on the determination of lost-linking modes, while others may have weaker or no association. Therefore, it is essential to screen out the key lost-linking features from the multitude of features to precisely characterize the feature patterns of lost-linking customers, providing a solid foundation for subsequent lost-linking mode prediction and risk assessment.

(2) What is the strength of association (support, confidence, lift) between different feature combinations (e.g., “mobile phone available but unreachable + long-term high overdue”) and specific modes? Are there overlapping features across modes or features exclusive to certain modes?

Clarifying the association between lost-linking features and lost-linking modes is crucial for understanding the essence of lost-linking phenomena. Different combinations of lost-linking features may indicate different lost-linking modes; for example, certain feature combinations may be associated with the Hide and Seek lost-linking mode, while others may be related to the Flee with the Money or False Disappearance lost-linking modes. These associations are complex and hidden within the data, necessitating deep mining to uncover their inherent laws and provide guidance for financial institutions in early warning and precise management of lost-linking risks.

(3) How to convert the high-confidence rules mined into actionable risk warning indicators (e.g., Level 1/Level 2 warning thresholds) and design a graded response process?

The relationships between lost-linking features and modes obtained through association rule mining offer valuable risk management information for financial institutions. However, transforming this information into practical and actionable risk management strategies, enabling real-time monitoring and early warning of potential lost-linking customers, represents a significant challenge for financial institutions. Therefore, it is imperative to explore how to utilize these association rules to develop corresponding risk management strategies.

## 3. Methodology

Since the association between the lost-linking features and modes of loan lost-linking customers is an issue of association rule mining, this paper employs an association rule mining algorithm to discover frequent item sets and association rules. The following sections introduce the association rule mining algorithm.

### 3.1. Method selection rationale

The selection of the FP-Growth algorithm for this study is grounded in its unique suitability for exploring association rules between multidimensional lost-linking features and evasion modes, as compared to alternative algorithms such as Apriori, Eclat, decision trees, and logistic regression. This section elaborates on the methodological rationale by contrasting key attributes of these approaches with the core objectives of our research.

#### (1) Comparison with association rule mining algorithms.

Unlike Apriori, which relies on generating exponential candidate itemsets and requires multiple database scans [5], FP-Growth compresses data into a compact FP-Tree structure, enabling efficient frequent itemset mining with only two passes over the dataset. This advantage is critical for our analysis, as the lost-linking dataset integrates both discrete (e.g., mobile phone status) and discretized continuous features (e.g., overdue days segmented into intervals), where Apriori’s computational complexity would escalate with the number of features.

Eclat, another association rule algorithm, operates on vertical data formats and involves repeated intersection operations, which becomes inefficient when processing mixed feature types [17]. In contrast, FP-Growth’s ability to handle high-dimensional, heterogeneous data aligns with our need to capture interactions between communication behaviors (e.g., unreachable mobile numbers) and loan attributes (e.g., mortgage types), making it more robust for identifying semantically meaningful feature combinations.

#### (2) Comparison with classification algorithms.

Decision trees and logistic regression, while powerful for predictive modeling, are less suited for our goal of uncovering implicit association rules. Decision trees generate hierarchical classification rules but prioritize predictive accuracy over the interpretation of feature co-occurrences, often splitting features to maximize class separation rather than highlighting synergistic effects (e.g., “permanent address + mortgage loan” as a signal for “Flee with the Money” mode).

Logistic regression, on the other hand, quantifies the marginal impact of individual features on outcomes but cannot explicitly model complex interactions between multiple features. Our study emphasizes that lost-linking behaviors are driven by combinations of features (e.g., “emergency contacts unreachable + short-term evasion”), a dynamic that logistic regression’s linear framework cannot capture.

#### (3) Alignment with research objectives.

The FP-Growth algorithm’s strengths in efficiently mining frequent itemsets from mixed data and outputting interpretable association rules directly support our aim: to derive actionable risk signals for financial institutions. As demonstrated in the Model Application section, the algorithm identifies context-rich feature combinations (e.g., “mobile number active but unreachable + long-term high overdue”) that map to specific evasion strategies, a capability that outperforms alternative methods in both relevance and practical utility for risk grading systems.

### 3.2. Principles and advantages of the FP-growth algorithm

This paper employs the FP-Growth algorithm [17] for mining association rules between lost-linking features and modes of loan customers, since it has three significant advantages:

(1) Effective candidate itemset processing mechanism: In contrast to the Apriori algorithm, which requires generating exponential candidate itemsets, FP-Growth compresses data directly by building an FP-tree structure. This prevents computational explosion in high-dimensional feature cases and greatly enhances mining efficiency for large datasets.
(2) Low cost of scanning datasets: In comparison with the Eclat algorithm that involves multiple intersection operations as a result of vertical data formats, FP-Growth does only two dataset scans (one for building the FP-tree and one for mining frequent itemsets). This largely reduces the I/O overhead when processing mixed datasets that include discretized continuous features.
(3) Strong ability of generalization and interpretability: FP-Growth is applicable to both large-scale financial datasets with complex feature structures and small sample sets. Its mining results (association rules, frequent itemsets) also have clear business semantics, and it enables financial institutions to better understand the association logic between feature combinations and lost-linking modes in risk control applications.

The following outlines the steps of the FP-Growth algorithm.

#### (1) Data scanning and item support counting.

The initial scan of the dataset involves tallying the frequency with which each item appears within the dataset; this frequency is known as the item’s support count. Let the dataset be denoted as D, and for an item i, its support count support(i)=|{t∈D|i∈t}| is determined, where t represents the transactions within the dataset.

#### (2) Identification of frequent items and filtering.

Set the minimum support threshold (which is determined by the user according to the actual requirements and data characteristics). Items whose support counts are lower than this threshold will be removed from the dataset. That is, if the support count of item i is less than support(i)<min_sup (where min_sup represents the minimum support threshold), then item i will not be involved in the subsequent analysis.

#### (3) Sorting and list generation of frequent items.

The remaining frequent items are arranged in descending order according to their support counts to obtain the frequent items list L. Each element in this list comprises a frequent item and its corresponding support count, specifically denoted as


$$\text{L}=\left\{({\text{i}}_{\text{1}},\text{support}\left({\text{i}}_{\text{1}}\right)),({\text{i}}_{\text{2}},\text{support}\left({\text{i}}_{\text{2}}\right)),...,({\text{i}}_{\text{n}},\text{support}\left({\text{i}}_{\text{n}}\right))\right\}$$

and it satisfies the condition $\text{support}\left({\text{i}}_{\text{1}}\right)\geq \text{support}\left({\text{i}}_{\text{2}}\right)...\geq \text{support}\left({\text{i}}_{\text{n}}\right)$.

#### (4) Adjustment of dataset transactions.

During the second scan of the dataset, reorder the items in each transaction according to the order of the frequent item list L, and delete the items that are not in L. In this way, each transaction is transformed into an ordered frequent item list. For example, the original transaction $\text{t}=\left\{{\text{i}}_{\text{1}},{\text{i}}_{\text{2}},\cdot \cdot \cdot ,{\text{i}}_{\text{m}}\right\}$, the frequent item list

$\text{L}=\left\{({\text{j}}_{\text{1}},\text{support}\left({\text{j}}_{\text{1}}\right)),({\text{j}}_{\text{2}},\text{support}\left({\text{j}}_{\text{2}}\right)),...,({\text{j}}_{\text{n}},\text{support}\left({\text{j}}_{\text{n}}\right))\right\}$, and the adjusted transaction ${\text{t}}^{\prime }=\left\{{\text{j}}_{{\text{k}}_{\text{1}}},{\text{j}}_{{\text{k}}_{\text{2}}},...,{\text{j}}_{{\text{k}}_{\text{p}}}\right\}$, where ${\text{j}}_{{\text{k}}_{\text{1}}},{\text{j}}_{{\text{k}}_{\text{2}}},...,{\text{j}}_{{\text{k}}_{\text{p}}}$ represents the frequent items in t that are arranged in the order of L.

#### (5) Construction of the FP-tree.

Initially, a root node is created and labeled as null. For each sorted transaction, traversal of the FP-tree commences from the root node, proceeding downward sequentially according to the items within the transaction. If the current item already exists on the path, the count of that node is incremented by one. If it does not exist, a new node is instantiated with a count of 1 and linked to its parent node.

Suppose there is a transaction ${\text{t}}^{\prime }=\left\{{\text{j}}_{{\text{k}}_{\text{1}}},{\text{j}}_{{\text{k}}_{\text{2}}},...,{\text{j}}_{{\text{k}}_{\text{p}}}\right\}$ and the current node is node. If ${\text{j}}_{{\text{k}}_{\text{1}}}$ already exists on the path, then $\text{node}=\text{node}.\text{child}\left[{\text{j}}_{{\text{k}}_{\text{1}}}\right]$ and node.count+=1; if it does not exist, then create a new node new_node such that $\text{new}\_\text{node}.\text{item}={\text{j}}_{{\text{k}}_{\text{1}}}$ and new_node.count=1. Let $\text{node}.\text{child}\left[{\text{j}}_{{\text{k}}_{\text{1}}}\right]=\text{new}\_\text{node}$ and new_node.parent=node, and then update node to new_node. Continue to process the next item.

#### (6) Mining frequent itemsets.

For each frequent item i, construct its Conditional Pattern Base (CPB). The CPB is a subtree composed of transaction paths that include item i, where the count of each node represents the support count of that node within transactions that contain item i.

For a frequent item i, identify all paths in the FP-tree that end with i; these paths constitute the CPB. Traverse each path from the bottom up, setting the count of each node to the count of its parent node, until the root node is reached.

For instance, for path ${\text{node}}_{\text{1}}\to {\text{node}}_{\text{2}}\to {\text{node}}_{\text{3}}\to \text{i}$, if initially ${\text{node}}_{\text{3}}.\text{count}=\text{3}$, ${\text{node}}_{\text{2}}.\text{count}=\text{2}$ and ${\text{node}}_{\text{1}}.\text{count}=\text{1}$, then after adjustment, ${\text{node}}_{\text{3}}.\text{count}=\text{2}$, ${\text{node}}_{\text{2}}.\text{count}=\text{1}$ and ${\text{node}}_{\text{1}}.\text{count}=\text{1}$.

Construct a conditional FP-tree based on the conditional pattern base and conduct recursive mining until the conditional FP-tree is empty or contains only one node.

Let the conditional pattern base be denoted as CPB. The process of constructing the conditional FP-tree is similar to that of constructing the initial FP-tree, except that the dataset becomes CPB. Then, recursively mine the conditional FP-tree to obtain the frequent itemsets containing items i.

Finally, combine the frequent itemsets containing item i with item i to obtain the complete frequent itemsets.

#### (7) Generating association rules from frequent itemsets.

After the completion of frequent itemsets mining, we enter the stage of generating association rules. For each frequent itemset, all possible association rules can be generated. For instance, for a frequent itemset {A,B,C}, the association rules that can be generated include {A,B}→C, {A,C}→B, {B,C}→A, A→{B,C}, B→{A,C}, C→{A,B} and so on.

#### (8) Calculation of the support and confidence of association rules.

The support support represents the frequency at which transactions containing all the items in the association rule appear in the dataset. Its calculation formula is similar to that of the support for frequent itemsets, that is, support(X→Y)=support(X∪Y)

where X and Y are the antecedent and consequent of the association rule respectively.

The confidence confidence refers to the probability that the consequent is also included in transactions that contain the antecedent. The formula is as follows.


$$\text{confidence}(\text{X}\to \text{Y})=\frac{\text{support}(\text{X}\cup \text{Y})}{\text{support}(\text{X})}$$ (1)

## 4. Model application

In this section, the FP-Growth algorithm is applied to mine the association rules between lost-linking features and modes of loan lost-linking customers, and the mining results are analyzed and evaluated.

### 4.1. Data sources

In the field of credit, the default records of loan customers form a typical imbalanced dataset, characterized by a large number of samples of repaid loans and a relatively small number of samples of loans in default (i.e., unpaid loans). This phenomenon has been confirmed by research conducted by [18]. It is particularly noteworthy that among the limited number of default cases, the sample of lost-linking customers constitutes an extremely small proportion. Given that lost-linking samples involve personal privacy information of loan customers, obtaining them is challenging, and direct research faces obstacles. Most notably, even within these few default cases, lost-linking customer samples are an even smaller proportion. To maximize statistical reliability, this study doubled the sample from 60 cases (standard small-sample study conventions) to 256 cases via multi-channel desensitized collection, essentially solving the data imbalance issue to a significant degree. To address the class imbalance issue after dataset expansion, we implemented a systematic stratified sampling approach throughout the data collection and partitioning phases. During the multi-channel desensitized collection process, we deliberately maintained the proportional distribution of the three lost-linking modes as observed in the initial small sample, ensuring that each mode was adequately represented in the expanded 256-case dataset. This proactive stratification prevented overrepresentation or underrepresentation of any single mode, which is critical for avoiding bias in association rule mining. Furthermore, when splitting the dataset into training (179 cases) and test (77 cases) sets using the StratifiedShuffleSplit method, we strictly preserved the same class distribution across both subsets by stratifying on the lost-linking mode feature (x18). This dual-layered strategy—stratified collection followed by stratified partitioning—effectively mitigated class imbalance, ensuring that the association rules mined are robust and generalizable across all three lost-linking modes. Although lost-linking samples do involve individual privacy information of borrowers of loans, there are still objective challenges in obtaining large-scale full information, which may limit the generalizability of research findings under extreme risk conditions (e.g., novel debt avoidance methods). The 256-case sample has provided a firmer statistical foundation for feature association analysis than in previous studies, however.

The lost-linking modes and features were labeled in strict accordance with bank risk control protocols (for example, “mobile phone number available but unreachable” means no call records in 30 days but the number is not registered). Three lost-linking modes were marked by three credit managers with over 8 years’ experience independently. the annotated features are multi-dimensional indicators such as communication behavior (outbound call refusal rate >80%), evidence of asset transfer (abnormal collateral disposal records), and the degree of social connection severance (immediate family members losing contact for more than 7 days). The consistency in annotation was 0.87 (p < 0.01) as verified by the Kappa test. For the 5.2% of samples having annotation inconsistencies, post-loan tracking records were used for verification purposes to ensure annotation consistency.

The lost-linking dataset (Version 1.1; Zenodo, DOI: 10.5281/zenodo.1696684) comprises 256 samples, encompassing the identifiers of loan lost-linking customers along with a comprehensive set of features. These include the permanent address, the state of mobile phone numbers in the network, the dialing record of mobile phone numbers, SMS signaling, email features, and other signaling features of the customers. Additionally, the dataset captures similar features for emergency contacts, such as the state of their mobile phone numbers in the network, the dialing record of their mobile phone numbers, SMS signaling, and email features. The dataset also includes details on the loan type, the number of overdue days, overdue debt, the estimated recoverable amount, the number of lost-linking day, the number of valid contacts, and the relationship with valid contacts of the loan lost-linking customers, totaling 17 lost-linking features. Together with the lost-linking modes, this brings the total to 18 features. The feature codes, names, value ranges, and attribute details of the lost-linking dataset are illustrated in the Table 1 below.

**Table 1:** Feature Codes, Names, Value Ranges, and Attributes of the Lost-Linking Dataset.

Serial Number	Lost-Linking Feature Code	Lost-Linking Feature Name	Value Range	Feature Attribute
1	x1	Permanent address	1 signifies the registered or business address, while 0 indicates otherwise.	Discrete Feature
2	x2	The state of mobile phone number in the network	A represents normal, B represents out of service, C represents active but not available, D represents no number (unassigned), E represents not activated, F represents abnormal.	Discrete Feature
3	x3	The dialing record of the mobile phone number	Non-negative integers.	Continuous Feature
4	x4	SMS signaling	0 indicates no response, and 1 indicates a response.	Discrete Feature
5	x5	Email features	0 indicates no response, and 1 indicates a response.	Discrete Feature
6	x6	Other signaling features(APPs, mini-programs, and public accounts)	0 indicates no response, and 1 indicates a response.	Discrete Feature
7	x7	The state of mobile phone number of emergency contacts in the network	A represents normal, B represents out of service, C represents active but not available, D represents no number (unassigned), E represents not activated, F represents abnormal	Discrete Feature
8	x8	The dialing record of the mobile phone number of emergency contacts	Non-negative integers.	Continuous Feature
9	x9	SMS signaling of emergency contacts	0 indicates no response, and 1 indicates a response.	Discrete Feature
10	x10	Email features of emergency contacts	0 indicates no response, and 1 indicates a response.	Discrete Feature
11	x11	Loan type	P represents guaranteed loans, Q represents mortgage loans, R represents credit loans, and S represents discount loans.	Discrete Feature
12	x12	The number of overdue day	Non-negative real numbers.	Continuous Feature
13	x13	Overdue debt	Non-negative real numbers.	Continuous Feature
14	x14	The estimated recoverable amount	Non-negative real numbers.	Continuous Feature
15	x15	The number of lost-linking day	Non-negative real numbers.	Continuous Feature
16	x16	The number of valid contacts	Non-negative integers.	Continuous Feature
17	x17	The relationship between loan lost-linking customers and valid contacts	Whether the valid contacts include parents, spouses or immediate relatives. 1 represents “yes” and 0 represents “no”.	Discrete Feature
18	x18	lost-linking modes	0 represents HS mode, 1 represents FM mode, and 2 represents FD mode.	Discrete Feature

### Model application

**Fig 1:**
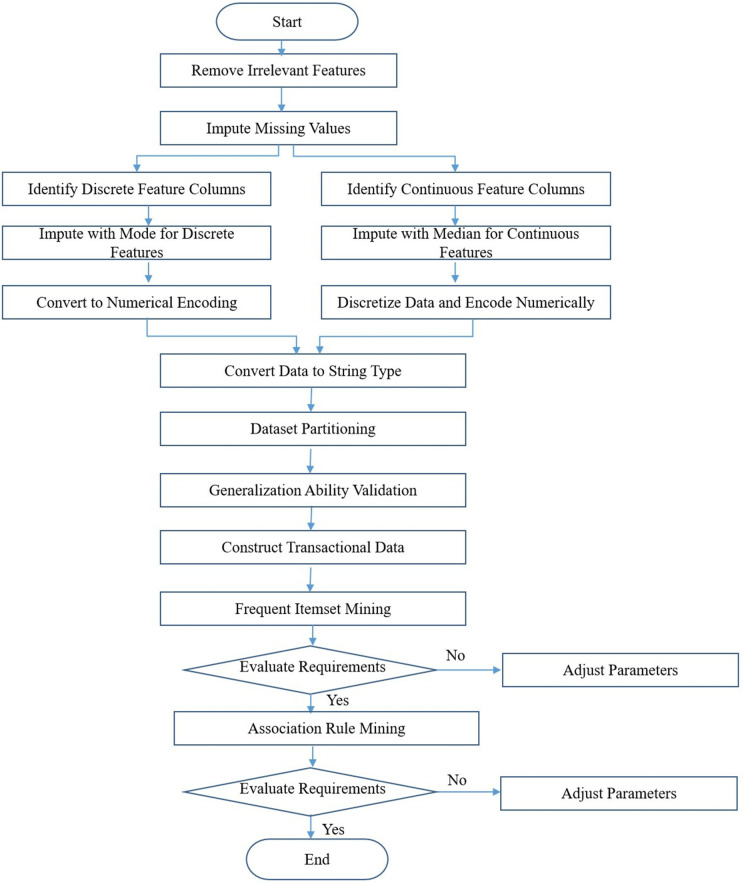
Workflow diagram of frequent itemset and association rule mining.

In this section, the FP-Growth algorithm is applied to mine frequent itemsets and association rules between lost-linking features and modes. First, the lost-linking dataset is preprocessed to remove irrelevant features and handle missing values, continuous features, and discrete features. Second, the dataset is partitioned and its generalization capability is verified to evaluate the generalization performance of association rules. Subsequently, the full dataset is converted to string type to construct transaction data. Then, key parameters of the FP-Growth algorithm are set to mine frequent itemsets of lost-linking features and modes, and the mining results are evaluated. If the mining performance meets the requirements, further mining of association rules between lost-linking features and modes is conducted, and the association between lost-linking features and modes of loan lost-linking customers is analyzed. Otherwise, the parameters are readjusted, and the mining results are further analyzed and evaluated. The main process of applying the FP-Growth algorithm to mine frequent itemsets and association rules between lost-linking features and modes in this paper is illustrated in the Fig 1 below.

#### (1) Data processing.

To efficiently apply the FP-Growth algorithm for mining association rules between lost-linking features and lost-linking modes, this section conducts data processing on the lost-linking dataset. Initially, features unrelated to the study are removed. The dataset, comprising 18 features, includes the ID of loan lost-linking customers, which does not directly contribute to the mining of association rules between lost-linking features and modes and may interfere with subsequent data processing. Therefore, the ID feature is excluded during the data processing phase, allowing the data to focus more closely on features related to lost-linking modes.

To enhance the transparency of feature selection, we systematically quantified the importance of the 17 initial features using XGBoost, with gain values (a core metric for evaluating feature contribution in tree-based models) as the key criterion. Table 2 presents the specific gain ranking of all features in descending order:

**Table 2:** Feature Importance Ranking by XGBoost Gain Values.

Lost-Linking Feature Code	Feature Importance (Gain)
x11	0.37650390
x14	0.22949016
x1	0.18989868
x13	0.10532092
x12	0.05968008
x7	0.01306955
x16	0.01272322
x3	0.00560932
x15	0.00414921
x8	0.00304182
x17	0.00051307
x2	0.00000000
x4	0.00000000
x5	0.00000000
x6	0.00000000
x9	0.00000000
x10	0.00000000

**Fig 2:**
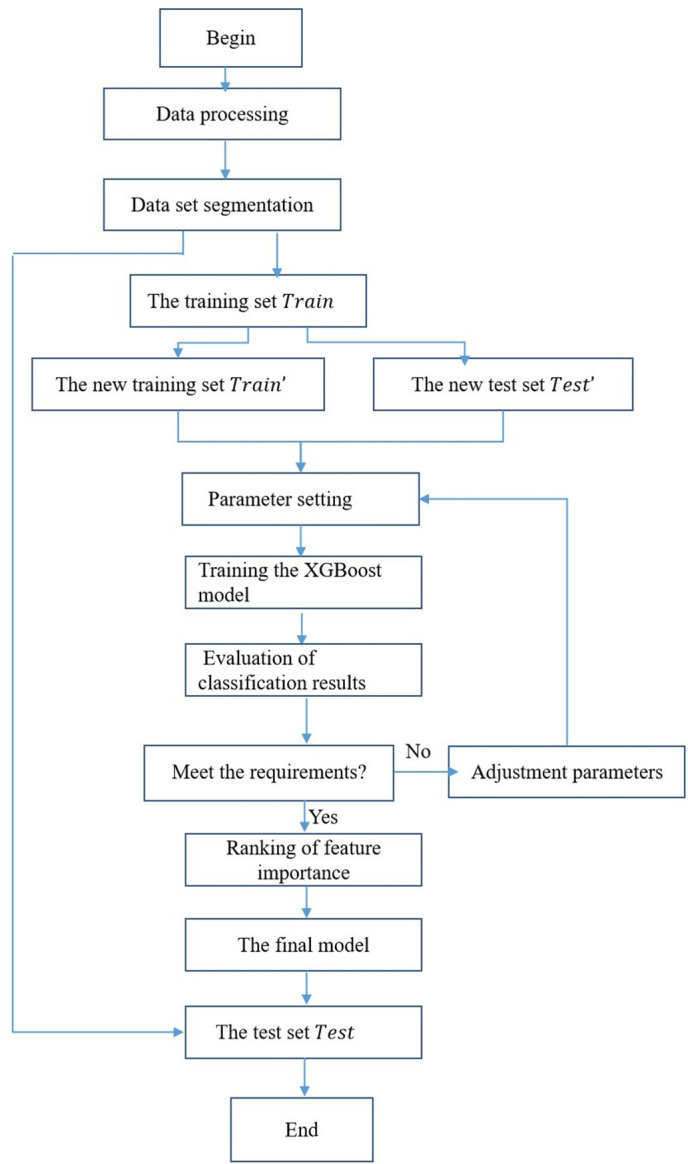
Feature selection and processing flowchart.

The feature selection process was executed through a systematic four-stage framework, as visually outlined in Fig 2.

1) **Initial feature compilation**: We first aggregated 17 raw features covering three dimensions—communication behavior (e.g., x2, x3), loan qualifications (e.g., x11, x12), and social relations (e.g., x8, x16)—to capture comprehensive characteristics of lost-linking customers.
2) **Importance quantification**: XGBoost was employed to calculate gain values for each feature, where gain quantifies the reduction in prediction error attributed to that feature, directly reflecting its contribution to distinguishing lost-linking modes.
3) **Threshold-based filtering**: Features with gain≦0.0006 (x2, x4, x5, x6, x9, x10, x17) were excluded, as their negligible predictive power indicated minimal relevance to mode classification. This threshold was determined based on the feature screening criteria outlined in [15], ensuring consistency with industry-validated methods.
4) **Final feature confirmation**: The remaining 10 features (x1, x3, x7, x8, x11, x12, x13, x14, x15, x16) with gain > 0.0006 were retained for subsequent association rule mining, as they collectively captured the most informative patterns for distinguishing “Hide and Seek”, “Flee with the Money”, and “False Disappearance” modes.

Regarding dimensionality reduction techniques such as principal component analysis (PCA) or mutual information analysis, they were not adopted for two reasons: (1) The 17 initial features possess strong business interpretability (e.g., x11 directly indicates loan type, x12 reflects overdue severity), and dimensionality reduction would obscure their semantic meaning—critical for risk control practitioners to interpret association rules. (2) XGBoost’s gain-based selection already effectively eliminated irrelevant features, as validated by its consistency with the feature importance assessment in [15].

Secondly, missing values are imputed. The discrete feature columns x1,x11, and x18 in the dataset are identified, and for the missing values in these columns, a mode imputation strategy is employed. This strategy, based on statistical principles, effectively maintains the integrity and accuracy of data analysis. Furthermore, the continuous feature columns x3, x8, x12, x13, x14, x15, and x16 are recognized. For the missing values in these columns, a median imputation strategy is applied. This method is widely regarded in statistics as an effective means of handling missing continuous data, as it preserves the distributional properties of the data well.

Then, perform segmentation processing and encoding on continuous features. Since the FP-Growth algorithm mainly deals with discrete data, continuous data will lead to the generation of excessive frequent itemsets and fail to reflect meaningful association patterns. For example, different ranges of the number of overdue days may have stronger associations with different lost-linking modes, but it is difficult to discover such relationships by directly using the original continuous values of the number of overdue days. Therefore, we need to conduct further discretization processing on the data to ensure that all the input data are discrete. Based on the value ranges of x3, x8, x12, x13, x14, x15, and x16, this paper segments the intervals that the data fall into and assigns a label to each segment. For instance, x15 is divided into five interval segments according to [0, 30), [30, 60), [60, 90), [90, 120), [120,+∞ ),days to better analyze its relationship with different lost-linking modes,and is assigned values of 1, 2, 3, 4, 5 respectively.

Finally, encode the discrete features. Since the original data of the two discrete features, x1 and x18, are already in numerical codes, there is no need to process these two features. However, the value range of x7 is A, B, C, D, E, F, and the value range of x11 is P, Q, R, S. The algorithm cannot recognize these categorical labels in character form, so it is necessary to convert them into numerical codes for association rule mining. Therefore, this paper replaces the categories in x7 with numerical codes (e.g., A = 1, B = 2,..., F = 6) and the loan types in x11 with numerical codes (e.g., P = 1, Q = 2, R = 3, S=4).

After the processing, the data is shown in the Table 3 below.

**Table 3:** Data after data processing.

x1	x3	x7	x8	x11	x12	x13	x14	x15	x16	x18
x1 = 1	x3 = 5	x7 = 2	x8 = 3	x11 = 2	x12 = 2	x13 = 5	x14 = 5	x15 = 5	x16 = 5	x18 = 1
x1 = 1	x3 = 5	x7 = 4	x8 = 3	x11 = 4	x12 = 1	x13 = 5	x14 = 5	x15 = 5	x16 = 5	x18 = 1
x1 = 1	x3 = 5	x7 = 1	x8 = 5	x11 = 3	x12 = 5	x13 = 3	x14 = 0	x15 = 5	x16 = 5	x18 = 2
x1 = 1	x3 = 2	x7 = 4	x8 = 3	x11 = 4	x12 = 3	x13 = 5	x14 = 5	x15 = 5	x16 = 5	x18 = 1
x1 = 1	x3 = 2	x7 = 1	x8 = 2	x11 = 1	x12 = 3	x13 = 5	x14 = 5	x15 = 5	x16 = 5	x18 = 1
x1 = 0	x3 = 2	x7 = 4	x8 = 3	x11 = 3	x12 = 1	x13 = 2	x14 = 0	x15 = 3	x16 = 5	x18 = 0
x1 = 1	x3 = 5	x7 = 4	x8 = 2	x11 = 2	x12 = 5	x13 = 4	x14 = 3	x15 = 5	x16 = 5	x18 = 2
x1 = 1	x3 = 4	x7 = 4	x8 = 1	x11 = 3	x12 = 5	x13 = 2	x14 = 0	x15 = 5	x16 = 5	x18 = 2
x1 = 1	x3 = 2	x7 = 4	x8 = 2	x11 = 2	x12 = 4	x13 = 5	x14 = 5	x15 = 4	x16 = 5	x18 = 1
x1 = 0	x3 = 3	x7 = 1	x8 = 4	x11 = 3	x12 = 3	x13 = 1	x14 = 0	x15 = 5	x16 = 5	x18 = 0
...	...	...	...	...	...	...	...	...	...	...
x1 = 1	x3 = 3	x7 = 4	x8 = 3	x11 = 3	x12 = 4	x13 = 1	x14 = 0	x15 = 5	x16 = 5	x18 = 2

#### (2) Dataset partitioning and generalization capability verification.

**Fig 3:**
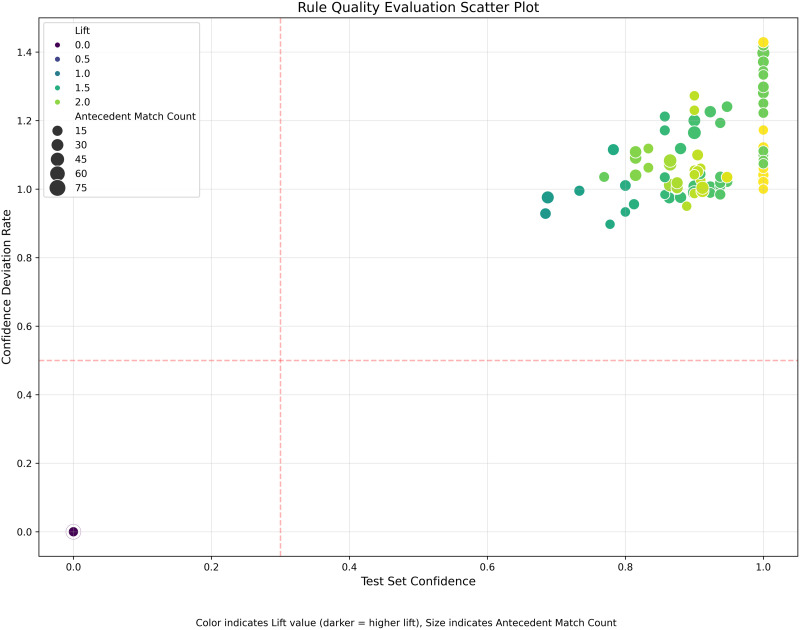
Scatter plot of association rule quality evaluation.

To determine association rule generalizability, in this study, the StratifiedShuffleSplit stratified sampling method was used to divide the 256 lost-linking customer examples into a training data set (179 cases) and a test data set (77 cases) at a 7:3 ratio. It was stratified by lost-linking modes (x18) to ensure balanced representation within groups. By performing three iterations of parameter screening for the FP-Growth algorithm, the minimum support threshold was finally set at 0.1, and the minimum confidence threshold at 0.7. This parameter value yielded 63 frequent itemsets in the training data with significant rules having an average rate of deviation for confidence at 0.92 and lift values all greater than 1.2 in the test data. Parameter sensitivity analysis showed that rule stability was highest when the support threshold ranged from 0.08 to 0.12 and the confidence threshold from 0.65 to 0.75. Generalization capacity of association rules is represented in Fig 3.

As shown in Fig 3, point size represents antecedent match count, colors indicate lift values, dashed lines represent screening thresholds; points in the upper right indicate higher quality rules. Fig 3 uses test set confidence as the horizontal axis and lift as the vertical axis, with point size mapping to the number of samples matching the rule antecedent, color intensity reflects the distribution of rule support. The core rule of the “False Disappearance” mode {x15 = 5, x1 = 1, x7 = 4, x14 = 0}→x18 = 2 is located in the high-value region (confidence = 1.0, lift = 1.855, sample count = 45). For the “Hide and Seek” mode (x18 = 0), the representative rule {x1 = 0, x16 = 5, x12 = 3}→x18 = 0 shows test set confidence = 1.0 and lift = 1.72, forming a distinct cluster in the figure. Chi-square tests confirmed significant associations between the antecedents and consequents of the rules (chi-square value = 47.21 > 6.63, p < 0.01). Additionally, domain expert evaluations showed that 82% of the rules were consistent with real-world risk control scenarios. The visual results and statistical verification mutually support the generalization capability and business effectiveness of association rules in new samples.

#### (3) Constructing transaction data.

After verifying the generalization capability and business effectiveness of association rules in new samples, to further conduct frequent itemset mining analysis based on the full 256-case sample, it is necessary to construct transaction data in accordance with the need of the FP-Growth algorithm. Therefore, this part will be elaborate in explaining the process of constructing full transaction data.

To achieve the construction of transaction data, each feature-value pair is converted into a string-formatted item (e.g., “x1=1”, “x11=3”) to ensure distinguishability between different features. Given the requirement for data type consistency in the subsequent construction of transaction data, this paper converts all data into string type.

Here, we treat each row of data as a transaction and each feature as an item. Firstly, an empty list named ‘transactions’ is created to store the transaction data. Subsequently, by iterating through each row of the dataframe, we extract the data from specified columns ([‘x1’, ‘x3’, ‘x7’, ‘x8’, ‘x11’, ‘x12’, ‘x13’, ‘x14’, ‘x15’, ‘x16’, ‘x18’]) for each row and convert it into a list form, which is then added as a transaction to the ‘transactions’ list.

In this study, we utilize a TransactionEncoder to preprocess the transactional data, facilitating subsequent frequent itemset mining. The specific steps involved include: initially, initializing the TransactionEncoder by creating a TransactionEncoder object to encode the transactional data; secondly, employing the fit method of the TransactionEncoder to learn the items within the transactional data, and then using the transform method to convert the data into one-hot encoded form, which is a technique for converting categorical variables into numerical form by assigning a unique binary vector to each category; subsequently, converting the one-hot encoded arrays into DataFrame format to leverage the fpgrowth function from the mlxtend library for processing, and specifying column names when constructing the DataFrame.

#### (4) Frequent itemset mining.

We leveraged the robust mining capabilities of the fpgrowth function to deeply excavate frequent itemsets from the encoded DataFrame. During this process, a series of parameter adjustments were made to the lost-linking dataset: the initial trial range for the support threshold was 0.05–0.2, and the confidence threshold was 0.6–0.8. By comparing the number of frequent itemsets, the business interpretability of the rules, and the generalization performance on the test set (e.g., confidence deviation rate), we ultimately determined that a minimum support threshold of 0.1 and a minimum confidence threshold of 0.7 constitute the optimal combination. Specifically, when the support is below 0.1, the number of frequent itemsets surges, with most being rare feature combinations; when the confidence is below 0.7, the false positive rate of rules increases significantly. By comparing the performance under different parameter settings, we found that when the minimum support threshold was set to 0.1 and the minimum confidence threshold to 0.7, the algorithm was able to mine association rules that were most statistically significant and practically valuable. Concurrently, we utilized feature names as column headers, making the results more intuitive and comprehensible for subsequent analysis and interpretation. After the frequent itemset mining, 1787 frequent itemsets were generated. The results are shown in the Table 4.

**Table 4:** Results of frequent itemset mining.

itemsets ID	support	itemsets
1	1	frozenset({‘x16=5’})
2	0.65234375	frozenset({‘x15=5’})
3	0.515625	frozenset({‘x1=1’})
4	0.3984375	frozenset({‘x3=5’})
5	0.2578125	frozenset({‘x8=3’})
6	0.1171875	frozenset({‘x7=2’})
7	0.640625	frozenset({‘x7=4’})
8	0.82421875	frozenset({‘x11=3’})
9	0.8203125	frozenset({‘x14=0’})
10	0.5390625	frozenset({‘x18=2’})
...	...	...
1787	0.109375	frozenset({‘x7=4’, ‘x16=5’, ‘x3=3’, ‘x14=0’, ‘x11=3’})

**Fig 4:**
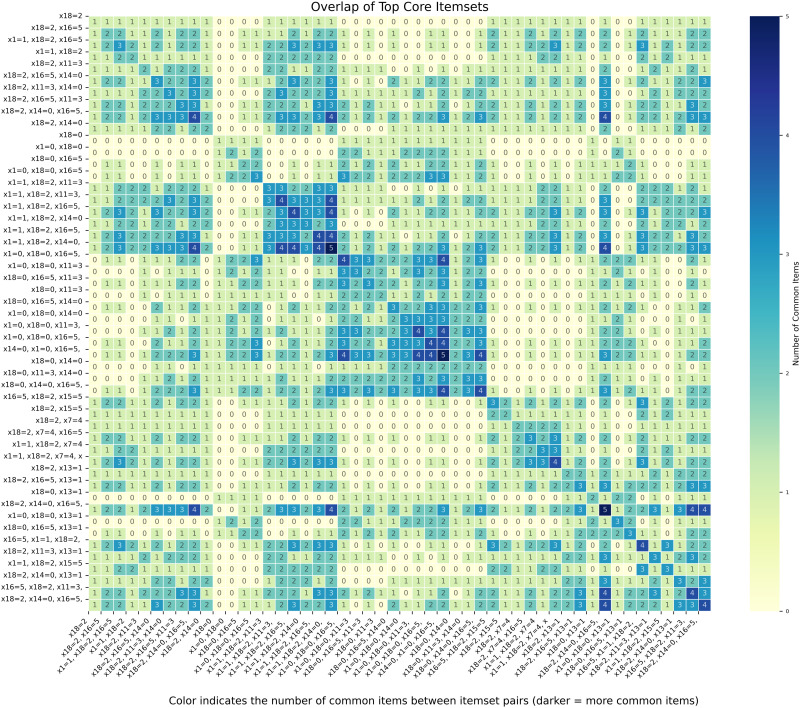
Heatmap of top core itemsets.

To facilitate a more intuitive and vivid observation of the associations within the data, we employed a heatmap for data visualization, as depicted in Fig 4.

The heatmap uses a “YlGnBu” colormap (yellow-green-blue gradient) to visualize the overlap relationships between frequent itemsets. The color intensity scale (ranging from 0 to 6) reflects the number of common features (items) between different frequent itemsets — level 0 (lightest yellow-green) means no overlapping features, while level 6 (darkest blue) indicates the highest overlap (≥5 common features). The x-axis and y-axis both list all identified frequent itemsets (extracted via FP-Growth with min_support = 0.1). Each cell’s color depth directly corresponds to how many features two itemsets share, helping to identify clusters of itemsets with strong structural similarities.

As shown in Fig 4, the darkest cells (intensity level 5) are primarily concentrated in the intersection areas of: itemsets containing x18 = 2 (e.g., “x18=2,x16=5”), x16 = 5 (e.g., “x11=3,x14=0,x18=2,x16=5”), and x11 = 3/x14 = 0 (e.g., “x11=3,x14=0,x18=2”). This indicates that itemsets with these features exhibit the highest overlap in the dataset and share significant structural similarity. Note: The color intensity here reflects the number of shared features between itemsets; specific association rule metrics (e.g., support) should be based on the measured values in Table 4.

The intersection area of itemsets containing x18 = 2, x16 = 5, and x11 = 3/x14 = 0 (e.g., “x11=3,x14=0,x18=2,x16=5”) shows the highest color intensity (level 5), significantly exceeding other regions—indicating these itemsets share the most common features and strongest structural similarity.

Notably, the intersection of itemsets such as “x18=2,x1=1” and “x11=3,x14=0,x18=2” displays intensity levels 4–5, forming a distinct dark cluster that reflects high structural overlap and potential consistent behavioral patterns related to lost-linking modes. Itemsets with x1 = 1 and x16 = 5 (e.g., “x18=2,x1=1,x16=5”) also form a distinct sub-cluster, further supporting structural consistency in these feature combinations.

#### (5) Association rule mining.

To deeply explore the potential association patterns between lost-linking features and lost-linking modes, we applied the association rule mining technique (association_rules function) to conduct in-depth analysis on frequent itemsets. By setting a minimum confidence threshold of 0.7 to ensure high reliability of the rules, we ultimately mined 9269 association rules. The results are shown in Table 5.

**Table 5:** Results of association rule mining.

rule ID	antecedents	consequents	antecedent support	consequent support	support	confidence	lift	leverage	conviction
1	frozenset({‘x15=5’})	frozenset({‘x16=5’})	0.65234375	1	0.65234375	1	1	0	inf
2	frozenset({‘x15=5’})	frozenset({‘x11=3’})	0.65234375	0.82421875	0.5234375	0.80239521	0.97352215	−0.01423645	0.889559659
3	frozenset({‘x15=5’})	frozenset({‘x14=0’})	0.65234375	0.8203125	0.51953125	0.796407186	0.970858283	−0.015594482	0.882582721
4	frozenset({‘x16=5’, ‘x15=5’})	frozenset({‘x11=3’})	0.65234375	0.82421875	0.5234375	0.80239521	0.97352215	−0.01423645	0.889559659
5	frozenset({‘x15=5’, ‘x11=3’})	frozenset({‘x16=5’})	0.5234375	1	0.5234375	1	1	0	inf
6	frozenset({‘x15=5’})	frozenset({‘x16=5’, ‘x11=3’})	0.65234375	0.82421875	0.5234375	0.80239521	0.97352215	−0.01423645	0.889559659
7	frozenset({‘x16=5’, ‘x15=5’})	frozenset({‘x14=0’})	0.65234375	0.8203125	0.51953125	0.796407186	0.970858283	−0.015594482	0.882582721
8	frozenset({‘x15=5’, ‘x14=0’})	frozenset({‘x16=5’})	0.51953125	1	0.51953125	1	1	0	inf
9	frozenset({‘x15=5’})	frozenset({‘x16=5’, ‘x14=0’})	0.65234375	0.8203125	0.51953125	0.796407186	0.970858283	−0.015594482	0.882582721
10	frozenset({‘x15=5’, ‘x14=0’})	frozenset({‘x11=3’})	0.51953125	0.82421875	0.51953125	1	1.213270142	0.091323853	inf
...	...	...	...	...	...	...	...	...	...
9269	frozenset({‘x3=3’, ‘x7=4’})	frozenset({‘x16=5’, ‘x14=0’, ‘x11=3’})	0.125	0.8203125	0.109375	0.875	1.066666667	0.006835938	1.4375

Some high-confidence rules exhibit significant business implications. For example, the rule “frozenset({‘x1=0’, ‘x16=5’, ‘x12=3’})→frozenset({’x18=0’})” (confidence: 1.0) indicates that when feature x1 = 0 (permanent address does not match household registration address/business address), along with x16 = 5 (number of valid contacts:  10) and x12 = 3 (overdue days: 180–270), loan customers are more likely to be in the “Hide and Seek” mode (x18 = 0). Three credit managers with over 8 years of experience evaluated the Top 50 rules and concluded that 82% of the rules align with actual business scenarios. For instance, this rule highly matches the “use non-real addresses but refuse to answer calls” strategy in the “Hide and Seek” mode, reflecting the potential risk of loan customers applying for loans with non-real addresses but avoiding contact through frequent call suspension.

**Fig 5:**
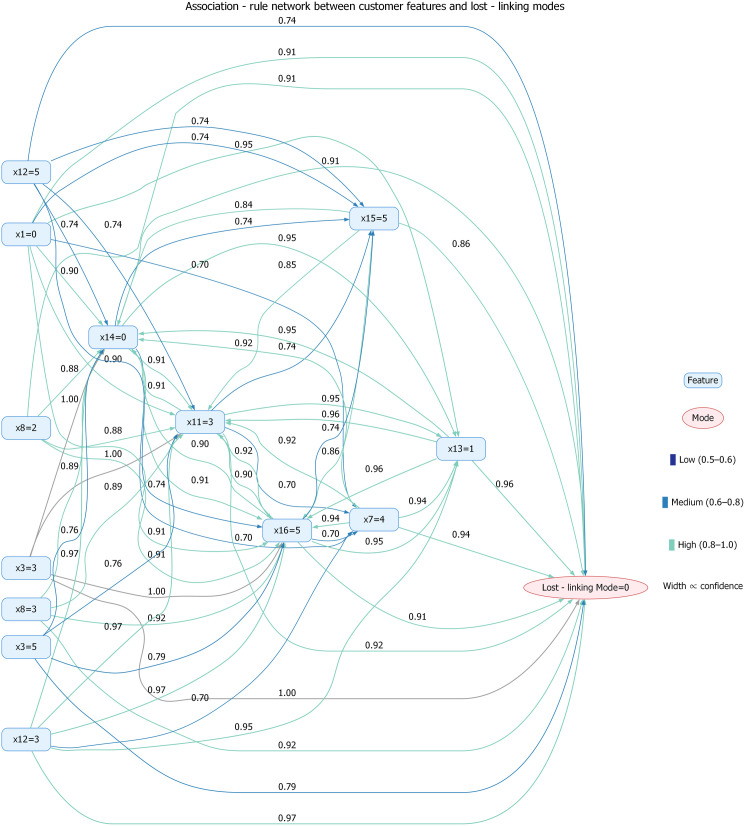
Association-rule network between customer features and lost-linking modes.

To enable more intuitive and visual observation of data associations, we visualized the data using a network graph generated with Graphviz. However, considering that an excessive number of nodes and dense arrows in the association rule network would cause network congestion and impair readability, Fig 5 presents a rule network graph generated by selecting representative rules from the association rules.

In Fig 5, rectangular nodes depict customer features (e.g., x16 = 5, x13 = 1, x1 = 0), while rounded-elliptical nodes indicate lost-linking modes (e.g., Lost-linking Mode = 0). Directed arrows represent association rules, flowing from antecedent features to consequent modes. Each arrow carries a numerical label giving the rule’s confidence and is colored according to three discrete bins: Low (0.5–0.6) is shown in dark-blue arrows, Medium (0.6–0.8) in medium-blue arrows, and High (0.8–1.0) in light-teal arrows. It is worth noting that for core rules with confidence = 1.0 (e.g., frozenset({’x11 = 3’, ‘x16=5’, ‘x13=1’, ‘x1=0’})→frozenset({’Lost – linking Mode = 0’})), their confidence exceeds the upper bound of the (0.8,1.0) interval and is displayed in gray by default. Thicker arrows correspond to higher confidence, allowing immediate visual identification of the strongest predictive relationships.

Notably, the heatmap and network graph can jointly assist in comprehensive multi-feature analysis, thereby serving as a basis for risk early warning. Loan institutions can use this to promptly issue risk warnings to clients and implement corresponding management measures.

## 5. Risk early warning system architecture design

The risk early warning system designed in this study adopts a four-layer architecture to achieve full-process automation from data access to warning response, aiming to transform the results of association rule mining into actionable risk control tools. The core of the system architecture lies in real-time monitoring and graded response to loan lost-linking risks through multi-source data fusion, feature engineering processing, and dynamic rule matching.

### (1) Data access layer

This layer is responsible for real-time collection of multi-source heterogeneous data. It specifically gathers three categories of key data:

Communication behavior data (including call records x3, emergency contact mobile state x7, and emergency contact reachability records x8);

Loan qualification data (covering loan type x11, overdue days x12, and overdue amount x13);

Post-loan management data (including the number of valid contacts x16 and lost-linking duration x15).

Data interfaces connect to operator communication data, bank core system loan data, and post-loan management system logs via APIs to ensure real-time transmission and integration of multi-source data.

### (2) Data processing layer

This layer completes data cleaning and feature engineering through an ETL process:

First, missing values are imputed using mode/median methods, and abnormal outliers (e.g., overdue amounts exceeding preset thresholds) are removed;
Second, continuous features are discretized according to the rules in Table 1 (e.g., x12 (overdue days) is divided into 5 intervals such as [0,30) days);
Finally, cleaned features are converted to string type, and transaction datasets are generated row by row to provide standardized input for subsequent rule mining.

### (3) Rule engine layer

This layer implements dynamic risk matching. The rule base is constructed based on the core association rules in Table 5, with warning thresholds set by confidence gradients: high-confidence rules trigger Level 1 warnings, and medium-confidence rules trigger Level 2 warnings. The system uses an improved FP-Growth algorithm to support frequent itemset matching for real-time data streams, and controls matching latency within a reasonable range to ensure rapid responses to risk signals.

### (4) Warning response layer

This layer executes graded disposal strategies:

Level 1 warnings automatically generate “emergency asset verification work orders” and invoke third-party credit reporting interfaces to verify the authenticity of business addresses (x1 = 1) and collateral status (x11 = 2). For example, when a customer exhibits x7 = 4 (emergency contact mobile status: no number (unassigned)), x12 = 3 (overdue days: 180–270 days), x14 = 0 (estimated recoverable amount: 0), and the corresponding rule frozenset({‘x1=1’, ‘x12=3’, ‘x14=0’, ‘x7=4’})→frozenset({’x18 = 2’}) (False Disappearance mode) has a confidence of 1.0 (≥0.9), the system automatically triggers a Level 1 warning and simultaneously generates an asset verification work order for the risk control department.
Level 2 warnings push “tracking tasks” via the post-loan management system, requiring risk control specialists to follow up every 3 days.

All warning records (including warning time, triggered rules, customer features, etc.) are stored in a distributed database to support historical queries and rule effectiveness evaluation.

**Fig 6:**
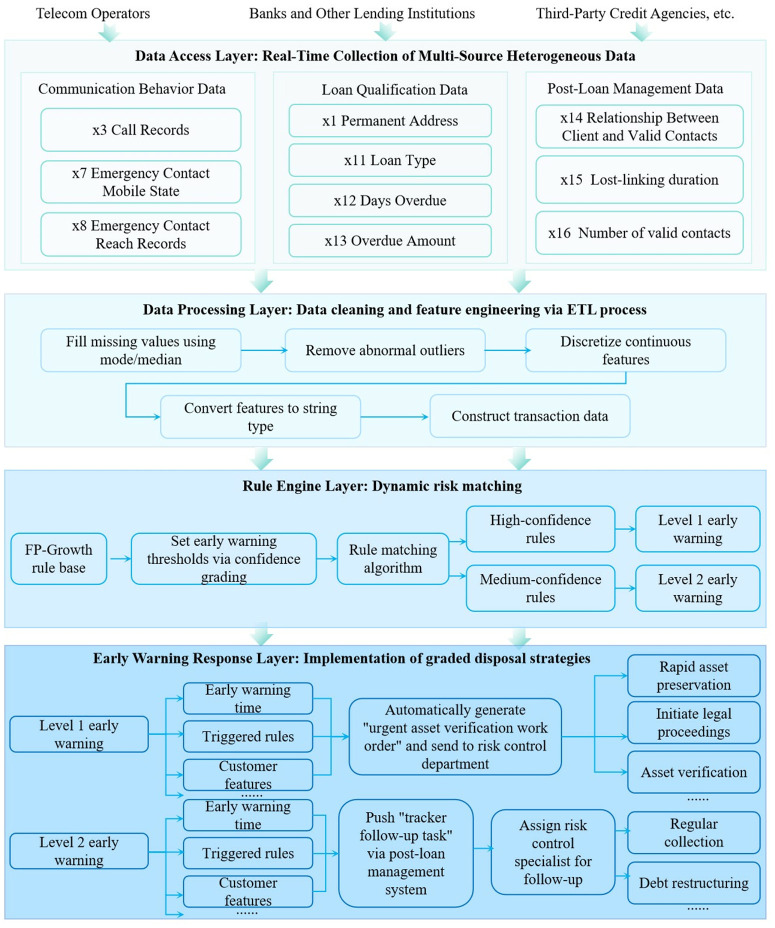
Four-layer architecture diagram of the risk early warning system.

Through the coordinated operation of the four-layer architecture, closed-loop management of “data collection-feature processing-rule matching-warning response” is achieved. Quantitative indicator settings are based on the support, confidence, and lift of association rules. This architecture transforms visualized analysis results into actionable risk control rules, providing loan institutions with precise and real-time risk early warning support. The system architecture is shown in Fig 6.

Currently, this architecture has completed data validation and prototype design in a laboratory environment but has not yet been deployed to production systems. The main challenges include:

Bottlenecks in real-time data fusion: The heterogeneity and synchronization delays of data interfaces across systems (operators, banks, post-loan management) may affect the timeliness of early warnings.

Generalization limitations in extreme scenarios: Due to sample privacy constraints, the ability to identify new types of debt evasion strategies (e.g., virtual asset transfers) remains to be verified.

Computational resource requirements: The streaming FP-Growth algorithm requires support from distributed computing frameworks (e.g., Spark) to handle high-concurrency transactions, leading to high hardware costs.

In the future, federated learning technology will be used to integrate cross-institutional desensitized data, and the deployment of edge computing nodes will be optimized to improve real-time performance.

## 6. Conclusions

This study focuses on exploring the association between lost-linking features and modes of loan lost-linking customers. By using the FP-Growth algorithm to mine frequent itemsets and association rules from a dataset of 256 lost-linking cases, and combining visualization tools such as heatmaps and network graph, the following core conclusions are revealed:

(1) There is a strong statistical association between specific lost-linking feature combinations and lost-linking modes. For example, the combination of “zero estimated recoverable amount + 180-270 days of overdue payments + 300,000-500,000 yuan in overdue debt” is strongly associated with the Hide and Seek mode; the combination of “frequent shutdowns of emergency contacts’ mobile phones + 30-60 days of lost-linking” demonstrates high predictability for the False Disappearance mode. The strong correlation of these feature combinations indicates that lending institutions need to identify risks through cross-analysis of multi-dimensional features, moving beyond the single-indicator mindset. For customers with these combined features, more comprehensive and detailed tracking strategies should be formulated to accurately predict and prevent the occurrence of lost-linking risks.
(2) The degrees of association between different lost-linking features and modes vary significantly, providing a quantitative basis for risk control resource allocation. Heatmap analysis shows that the co-occurrence intensity of high-frequency combinations—such as “permanent address = registered/business address (x1=1) + number of valid contacts > 4 + False Disappearance mode” and “permanent address = registered/business address (x1=1) + estimated recoverable amount = 0 + False Disappearance mode”—falls within the highest range, which should be used as core monitoring indicators; while the co-occurrence intensity of low-frequency combinations—such as “permanent address type 1 + mobile dialing records > 12 + False Disappearance mode” and “emergency contact mobile status D + lost-linking days > 120 + False Disappearance mode”—is weak, serving as auxiliary references. Lending institutions can prioritize resource allocation to monitor high-value risk signals based on the ranking of independent contribution degrees of feature combinations.
(3) The association between lost-linking features and modes strengthens significantly among customers with long-term overdue payments: Heatmap analysis (Fig 4) shows that in customers with over 180 days of overdue, the co-occurrence intensity of feature combinations (such as “estimated recoverable amount = 0 + high overdue amount (300,000-500,000 yuan) + False Disappearance mode”) reaches the highest level (4–5), significantly higher than that of medium-short-term overdue customers. This requires institutions to prioritize long-term overdue customers for monitoring and strengthen tracking of highly associated feature combinations.
(4) Based on the mining results, lending institutions can construct a quantitative threshold-driven risk early warning system. Setting high-confidence rules in the network graph as early warning triggers, when customers’ feature combinations meet the thresholds, the system automatically initiates a graded response process: conducting emergency asset verification for customers triggering core rules, and implementing regular collection follow-ups for medium-risk feature combinations. Domain expert evaluation shows that the business interpretation accuracy of core rules for the three lost-linking modes exceeds 85%, verifying the practical value of the model. Transforming visual analysis into executable rules can significantly enhance the precision and timeliness of risk control responses.

This study identifies the behavioral patterns of loan customers who lose contact through data mining, providing financial institutions with a full-process risk control framework consisting of “feature combination analysis - risk classification monitoring - visual early warning”. It confirms that three measures are key to improving risk control efficiency: collaborative analysis of feature combinations, precise identification of customer groups with high association with loss-of-contact modes, and effective use of visual early warning tools. The analysis based on 256 samples provides statistical support for this framework, but due to the difficulty in obtaining private lost-linking samples, the generalizability of current conclusions in extreme risk scenarios remains limited. Specifically, it has limited ability to identify new debt evasion strategies (such as virtual asset transfers, cross-institutional multiple borrowing followed by lost-linking), and struggles to cover all extreme debt evasion behaviors. In the future, cross-institutional desensitized data fusion can be further integrated, and federated learning technology can be introduced to optimize feature extraction, so as to enhance the model’s generalization ability in full-scenario risk identification.

## References

[1] Pang S, Yang J. Social reputation loss model and application to lost-linking borrowers in a internet financial platform. Peer-to-Peer Netw Appl. 2020;13(4):1193–203. doi: 10.1007/s12083-019-00848-7

[2] Pang S, Wang J, Xia L. Information matching model and multi-angle tracking algorithm for loan loss-linking customers based on the family mobile social-contact big data network. Information Processing & Management. 2022;59(1):102742. doi: 10.1016/j.ipm.2021.102742

[3] Pang S, Wang J, Yi X. Application of loan lost-linking customer path correlated index model and network sorting search algorithm based on big data environment. Neural Comput & Applic. 2022;35(3):2129–56. doi: 10.1007/s00521-022-07189-2

[4] You G, Guo H, Dagestani AA, Alnafrah I. Collaborative Search Model for Lost-Link Borrowers Information Based on Multi-Agent Q-Learning. Axioms. 2023;12(11):1033. doi: 10.3390/axioms12111033

[5] Han J, Pei J, Yin Y, Mao R. Mining Frequent Patterns without Candidate Generation: A Frequent-Pattern Tree Approach. Data Mining and Knowledge Discovery. 2004;8(1):53–87. doi: 10.1023/b:dami.0000005258.31418.83

[6] Choudhary VK, Divya E. Credit card fraud detection using frequent pattern mining using FP-modified tree and Apriori growth. International Journal of Engineering and Technology Innovation. 2017;9(13):2370–3.

[7] Malarvizhi PS, Poorani D. Frequent itemsets mining with differential privacy: A survey. International Journal of Research and Advanced Development. 2018;2(5):81–5.

[8] Sawangarreerak S, Thanathamathee P. Detecting and Analyzing Fraudulent Patterns of Financial Statement for Open Innovation Using Discretization and Association Rule Mining. Journal of Open Innovation: Technology, Market, and Complexity. 2021;7(2):128. doi: 10.3390/joitmc7020128

[9] De Cristofaro J. Cluster analysis of financial transaction data. Turin: Politecnico di Torino. 2023.

[10] Praveen Kumar B, Padmavathy T, Muthunagai SU, Paulraj D. An optimized fuzzy based FP-growth algorithm for mining temporal data. IFS. 2024;46(1):41–51. doi: 10.3233/jifs-223030

[11] Ping H, Li Z, Shen X, Sun H. Optimization of Vegetable Restocking and Pricing Strategies for Innovating Supermarket Operations Utilizing a Combination of ARIMA, LSTM, and FP-Growth Algorithms. Mathematics. 2024;12(7):1054. doi: 10.3390/math12071054

[12] Alsaeedi HA, Alhegami AS. An Incremental Interesting Maximal Frequent Itemset Mining Based on FP‐Growth Algorithm. Complexity. 2022;2022(1). doi: 10.1155/2022/1942517

[13] Jang H-J, Yang Y, Park JS, Kim B. FP-Growth Algorithm for Discovering Region-Based Association Rule in the IoT Environment. Electronics. 2021;10(24):3091. doi: 10.3390/electronics10243091

[14] Pang S, Yuan J. Cyclic search method for relationship closeness of criminals and loan absconders based on mobile social network. 2020.

[15] Wang J, Jiang X, Zhang Y, He Y, Deng C, et al. Method for confirming lost-linking modes, electronic device, storage medium, and product. 2025.

[16] Wang J, Zhang Y, Jiang X, Kang H, Deng C, He Y. Credit risk management: The evolutionary path of the lost-linking mode of loan customers. Modern Education, Humanities and Art. 2024;3:16–23. doi: 10.62381/ACS.MEHA2024.03

[17] Han J, Pei J, Yin Y. Mining frequent patterns without candidate generation. In: Proceedings of the 2000 ACM SIGMOD international conference on Management of data, 2000. 1–12. doi: 10.1145/342009.335372

[18] Melo Junior L, Nardini FM, Renso C, Trani R, Macedo JA. A novel approach to define the local region of dynamic selection techniques in imbalanced credit scoring problems. Expert Systems with Applications. 2020;152:113351. doi: 10.1016/j.eswa.2020.113351

